# Posterior circulation collaterals as predictors of outcome in basilar artery occlusion: a sub-analysis of the BASICS randomized trial

**DOI:** 10.3389/fneur.2024.1360335

**Published:** 2024-03-28

**Authors:** Fabricio O. Lima, Felipe A. Rocha, Henrique C. Silva, Volker Puetz, Diederik Dippel, Ido van den Wijngaard, Charles Majoie, Albert J. Yoo, Wim van Zwam, Adson F. de Lucena, Diego De Almeida Bandeira, Martin Arndt, Kristian Barlinn, Johannes C. Gerber, Lucianne C. M. Langezaal, Wouter J. Schonewille, Octávio M. Pontes Neto, Francisco Antunes Dias, Sheila Ouriques Martins, Francisco José de A. Mont’Alverne

**Affiliations:** ^1^Neurology Service, Hospital Geral de Fortaleza, Fortaleza, Brazil; ^2^Neurointerventional Service, Hospital Geral de Fortaleza, Fortaleza, Brazil; ^3^Department of Neurology, Technical University Dresden, Dresden, Germany; ^4^Dresden Neurovascular Center, Technical University Dresden, Dresden, Germany; ^5^Erasmus MC University Medical Center, Rotterdam, Netherlands; ^6^Department of Neurology, Haaglanden Medical Center, The Hague, Netherlands; ^7^Academic Medical Center, Amsterdam, Netherlands; ^8^Texas Stroke Institute, Plano, TX, United States; ^9^Interventional Radiology Department, Maastricht University Medical Centre, Maastricht, Netherlands; ^10^Institute of Neuroradiology, Dresden Neurovascular Center, Universitätsklinik Dresden, Dresden, Germany; ^11^Department of Neurology, University Medical Center, Utrecht, Netherlands; ^12^Stroke Service, Neurology Division, Department of Neurosciences and Behavioral Sciences, Ribeirão Preto School of Medicine, University of São Paulo, Ribeirao Preto, Brazil; ^13^Department of Neurology, Hospital de Clinicas de Porto Alegre, Federal University of Rio Grande do Sul, Porto Alegre, Brazil

**Keywords:** basilar artery occlusion, posterior circulation stroke, endovascular treatment, diagnostic imaging, collateral circulation, treatment outcome, prognosis factors, reperfusion therapy

## Abstract

**Introduction and purpose:**

Basilar artery occlusion (BAO) is still one of the most devastating neurological conditions associated with high morbidity and mortality. In the present study, we aimed to assess the role of posterior circulation collaterals as predictors of outcome in the BASICS trial and to compare two grading systems (BATMAN score and PC-CS) in terms of prognostic value.

**Methods:**

We performed a sub-analysis of the BASICS trial. Baseline clinical and imaging variables were analyzed. For the imaging analysis, baseline CT and CTA were analyzed by a central core lab. Only those patients with good or moderate quality of baseline CTA and with confirmed BAO were included. Multivariable binary logistic regression analysis was used to test the independent association of clinical and imaging characteristics with a favorable outcome at 3 months (defined as a modified Rankin Score of ≤3). ROC curve analysis was used to assess and compare accuracy between the two collateral grading systems.

**Results:**

The mean age was 67.0 (±12.5) years, 196 (65.3%) patients were males and the median NIHSS was 21.5 (IQR 11–35). Median NCCT pc-ASPECTS was 10 (IQR10-10) and median collateral scores for BATMAN and PC-CS were 8 (IQR 7–9) and 7 (IQR 6–8) respectively. Collateral scores were associated with favorable outcome at 3 months for both BATMAN and PC-CS but only with a modest accuracy on ROC curve analysis (AUC 0.62, 95% CI [0.55–0.69] and 0.67, 95% CI [0.60–0.74] respectively). Age (OR 0.97, 95% CI [0.95–1.00]), NIHSS (OR 0.91, 95% CI [0.89–0.94]) and collateral score (PC-CS – OR 1.2495% CI [1.02–1.51]) were independently associated with clinical outcome.

**Conclusion:**

The two collateral grading systems presented modest prognostic accuracy. Only the PC-CS was independently associated with a favorable outcome at 3 months.

## Introduction

Basilar artery occlusion (BAO) accounts for approximately 10% of strokes due to large vessel occlusion (LVO) and is present in about 8% of patients with symptomatic vertebrobasilar territory ischemia (1–3). Technical developments during the past few years have improved the diagnosis and management of BAO significantly, even so it is still one of the most devastating neurological conditions associated with high morbidity and mortality even in those treated with mechanical thrombectomy (4–7).

Several clinical and imaging predictors of outcome after BAO, such as the National Institutes of Health Stroke Scale (NIHSS) score at admission, age, PC-ASPECTS, and recanalization have been identified in earlier studies (3, 8). Collaterals have been recognized to influence recanalization, reperfusion, hemorrhagic transformation, and subsequent neurological outcomes, and are increasingly assessed in daily clinical practice, in patients with anterior circulation strokes (9). In contrast, the presence of posterior circulation collaterals in BAO patients is a feature that is yet far from being regularly used in clinical practice.

The noninvasive nature of CTA and its wide and rapid availability makes it the preferred diagnostic modality to assess collateral status in the acute stage of BAO. Two main collateral grading system exist for the posterior circulation, the posterior circulation collateral score (PC-CS) and the Basilar Artery on Computed Tomography Angiography (BATMAN) score (10, 11). Only a few studies have examined the role of posterior circulation collaterals as a predictor of outcome and systematically compared the two grading systems (12, 13).

In the present study, we aimed to assess the role of posterior circulation collaterals as predictors of outcome in the BASICS trial and to compare the two systems in terms of prognostic significance.

## Methods

We performed a sub-analysis of the BASICS trial, a multicenter, open-label, international, randomized, controlled trial with blinded outcome assessment to test the efficacy and safety of endovascular therapy initiated within 6 h after the estimated time of basilar-artery occlusion with best medical therapy (14). The design of the trial has been described in detail elsewhere (15).

Baseline clinical variables included: age, sex, cardiovascular risk factors, baseline NIHSS, glycemia, blood pressure and IV rt-PA use. Stroke subtyping was done according to the TOAST classification and further dichotomized into cardioembolic and other (grouping large vessel atherosclerosis, other causes and uncertain). Estimated time of BAO, defined as time consistent with the clinical diagnosis of BAO on the judgment of the treating physician, was used to compute several time metrics including symptom onset-BAO, symptom onset-IV rtPA, symptom onset-EVT and BAO-recanalization. The primary outcome analyzed was a favorable clinical outcome, defined as modified Rankin score (mRS) ≤3 at 90 days.

For the imaging analysis baseline CT and CTA were analyzed by a central core lab. Patients with low quality baseline CTA were excluded from the analysis. Reperfusion was defined as a TICI2b-3 adjudicate by a central core lab. The pc-ASPECTS on NCCT and the pc-ASPECTS on CTA were quantified (16). All occluded vessel segments (including absence of posterior communicating arteries – Pcomms) were identified. Collaterals were graded according to two grading systems – the Basilar Artery on Computed Tomography Angiography (BATMAN) score and Posterior Circulation Collateral score (PC-CS).

The BATMAN Score is a 10-point semiquantitative scoring system based on CTA that was graded as follows: one point for each of the other segments giving a maximum score of 10: 1 point if either intracranial vertebral artery was patent; 1 point for each patent segment of the basilar artery, the proximal segment, extending from the vertebrobasilar junction to the origin of AICAs, the middle segment from the origins of AICAs to the origin of SCAs, and the rostral segment from the origin of SCAs to its rostral end; and 1 point for each patent P1 segment of PCA (10).

The PC-CS ranges from 0 to 10 and was graded as follows: each patent PICA, AICA, and SCA is allocated with 1 point. Identification of a PCoA is allocated with 1 point if its diameter is smaller than the ipsilateral P1 segment and 2 points if its diameter is equal or larger than the ipsilateral P1 segment. Fetal variants of the PCA, defined as a PCA arising from the anterior circulation and absence of P1 segments, are not included (11).

Both PC-PCs and BATMAN scores were dichotomized in good vs. poor collaterals as previously reported (10, 11). For PC-CS good collaterals was defined as a score ≥ 6. For the BATMAN score good collaterals was defined as a score ≥ 7. We compared PC-ASPECTS (on both NCCT and CTA) at baseline and day 1 between patient with good and poor collaterals.

### Statistical analysis

Categorical variables were reported as absolute numbers and percentages. Continuous variables were reported as mean ± standard deviation (SD) or median ± interquartile range (IQR). Differences between proportions were assessed by the chi-square test or the Fisher’s exact test as appropriate. Differences in continuous variables were assessed by the independent- samples T-test or the Mann–Whitney U test in case of non-normally distributed data. Univariable binary logistic regression was used to generate OR and 95% CI for the analysis of continuous variables. Odds ratios (OR) and 95% confidence intervals (CI) are reported in all tests. The Wilcoxon signed rank test was used to compared PC-ASPECTS (for both NCCT and CTA) at baseline and day 1 between patients with good and poor collaterals.

ROC curve analysis was used to assess and compare accuracy between the two collateral grading systems. The area under the curve and 95% CI was calculated and used to compare the two curves. Binary logistic regression analysis was used to test the independent association of clinical and imaging characteristics with the primary outcome (mRS at 90 days). The variables in the univariable analysis associated with the primary outcome with a *p*-value ≤0.2 were included in the first model. Variables with known association with the outcome (age, NIHSS, IV rt-PA and endovascular treatment) and the predictors of interest (collaterals score) were selected *a priori* to be included in the final model. To avoid overfitting, only those variables with a *p* value ≤0.1 in the first model were kept in the final model. To test if posterior circulation collaterals have an effect modification of mechanical thrombectomy on the outcome, an interaction term (collateral score x mechanical thrombectomy) was added in the regression models. A *p*-value <0.05 was considered statistically significant. IBM SPSS version 25.0 software was used to perform all the statistical analyses.

## Results

Of 300 patients, 196 (65.3%) were men, the median NIHSS was 21.5 (IQR 11–35) and the mean age was 67.0 (±12.5) years. Hypertension was the most common risk factor (175–58.3%). Large artery atherosclerosis was the most common identified stroke mechanism (96–32.0%) followed by undetermined (85–28.3%) and cardioemboc etiology (75–25.0%). Most patients were treated with IV r-tPA (237–79.0%). The median time from BAO to EVT and from BAO to recanalization was 205 (IQR 150–275) and 273 (IQR 206–353) minutes, respectively. Reperfusion was achieved in 63/88 (71.6%) (Table 1).

**Table 1:** Baseline characteristics of the study population and outcome at 3 months.

Baseline clinical characteristics	Total		mRS 0–3		mRS 4–6		OR (95% CI)	*p*-value
	*N* = 300		*N* = 123		*N* = 177			
Age (years), (mean – SD)	67.0	(12.5)	64.0	(12.8)	69.1	(11.8)	0.97 (0.95–0.99)	<0.001
NIHSS, (median – IQR)	21.5	(11–35)	12	7–20	31	18–37	0.91 (0.89–0.93)	<0.001
	**N**	**%**	**N**	%	**N**	**%**		
NIHSS ≥10	239	79.7	79	64.2	160	90.4	0.19 (0.10–0.35)	<0.001
NIHSS ≥20	161	53.7	31	25.2	130	73.4	0.12 (0.07–0.21)	<0.001
Sex (male)	196	65.3	81	65.9	115	65.0	0.96 (0.59–1.56)	0.87
Hypertension	175	58.3	65	52.8	110	62.1	0.69 (0.43–1.11)	0.13
Diabetes	65	21.7	17	13.8	48	27.1	0.43 (0.24–0.80)	0.01
Atrial fibrillation	66	21.9	23	18.7	43	24.3	0.72 (0.41–1.27)	0.25
Peripheral arterial disease	18	6.0	6	4.9	12	6.8	0.69 (0.25–1.90)	0.47
Smoking (current/former)	120	40.0	56*	45.5	64*	36.1	0.82 (0.47–1.42)	0.47
Alcohol (current/former)	103	34.3	50*	40.6	53*	29.9	0.88 (0.49–1.56)	0.66
Stroke (posterior circulation)	18	6.0	7	5.7	11	6.2	0.91 (0.34–2.42)	0.85
TIA (posterior circulation)	8	2.7	4	3.2	4	2.2	1.43 (0.35–5.82)	0.62
Stroke mechanism								
Large artery atherosclerosis	96	32.0	38	30.9	58	32.8	Ref.	
Cardioembolism	75	25.0	31	25.2	44	24.8	1.07 (0.58–1.99)	
Other	21	7.0	11	8.9	10	5.6	1.68 (0.65–4.34)	0.72
Undetermined	85	28.3	33	26.8	52	29.4	0.97 (0.53–1.76)	
IV rtPA	237	79.0	102	82.9	135	76.3	1.51 (0.84–2.71)	0.16
Endovascular treatment	154	51.3	68	55.3	86	48.6	1.31 (0.82–2.08)	0.25
Reperfusion (TICI 2b/3)	63/88	71.6	30/41	73.2	33/47	70.2	1.16 (0.46–2.94)	0.76
**Time metrics (minutes)**	**Median**	**IQR**	**Median**	**IQR**	**Median**	**IQR**		
Symptom onset-BAO	0	0–90	0	0–49	0	0–116	1.0 (0.99–1.00)	0.03
Symptom onset-IV rtPA	130	90–202	129	83–197	134	100–207	0.9 (0.99–1.00)	0.12
Symptom onset-EVT	265	198–372	240	196–346	295	198–408	1.00 (1.00–1.00)	0.30
BAO-EVT	205	150–275	210	141–260	205	160–292	1.00 (0.99–1.00)	0.55
BAO-recanalization	273	200–345	272	197–355	274	204–339	1.00 (1.00–1.00)	0.24

Two hundred and fifty-two patients had a confirmed BAO according to the central core lab. Two hundred and thirty-seven patients were analyzed according to their baseline imaging characteristics. Median pc-ASPECTS was high in both NCCT (10; IQR10-10) and in CTA baseline imaging (10; IQR 8-10). Location of occlusion was roughly equal in all segments of the basilar artery (proximal – 79/33.3%; middle – 77/32.5% and distal – 81/34.2%). Median collateral scores for BATMAN and PC-CS were 8 (IQR 7–9) and 7 (IQR 6–8) respectively (Table 2).

**Table 2:** Baseline imaging characteristics and outcome at 3 months.

Baseline imaging characteristics	Total		mRS 0–3		mRS 0–4		OR (95% CI)	*p*-value
	*N* = 237		*N* = 95		*N* = 142			
	Median	IQR	Median	IQR	Median	IQR		
pc-ASPECTS (NCCT)	10	10–10	10	10–10	10	9–10	1.48 (1.01–2.17)	0.04
pc-ASPECTS (CTA)	10	8–10	10	8–10	10	8–10	1.26 (1.05–1.52)	0.15
Length BAO (mm)	11.0	6.5–18.0	8.0	5.5–14.0	13.5	8.4–20.0	0.94 (0.90–0.97)	<0.001
Collaterals								
BATMAN score	8	7–9	8	8–9	8	7–9	1.45 (1.16–1.81)	0.001
PC-CS	7	6–8	8	7–9	7	6–8	1.44 (1.22–1.72)	<0.001
	**N**	**%**	**N**	%	**N**	**%**		
Location of BAO								
Proximal basilar artery	79	33.3	23	24.2	56	39.4	ref	ref
Middle basilar artery	77	32.5	26	27.4	51	35.9	1.24 (0.63–2.44)	0.53
Distal basilar artery	81	34.2	46	48.4	35	24.6	3.20 (1.66–6.16)	<0.001
PCA occlusion	95	40.1	35	36.8	60	42.3	0.78 (0.47–1.36)	0.40

**Figure 1:**
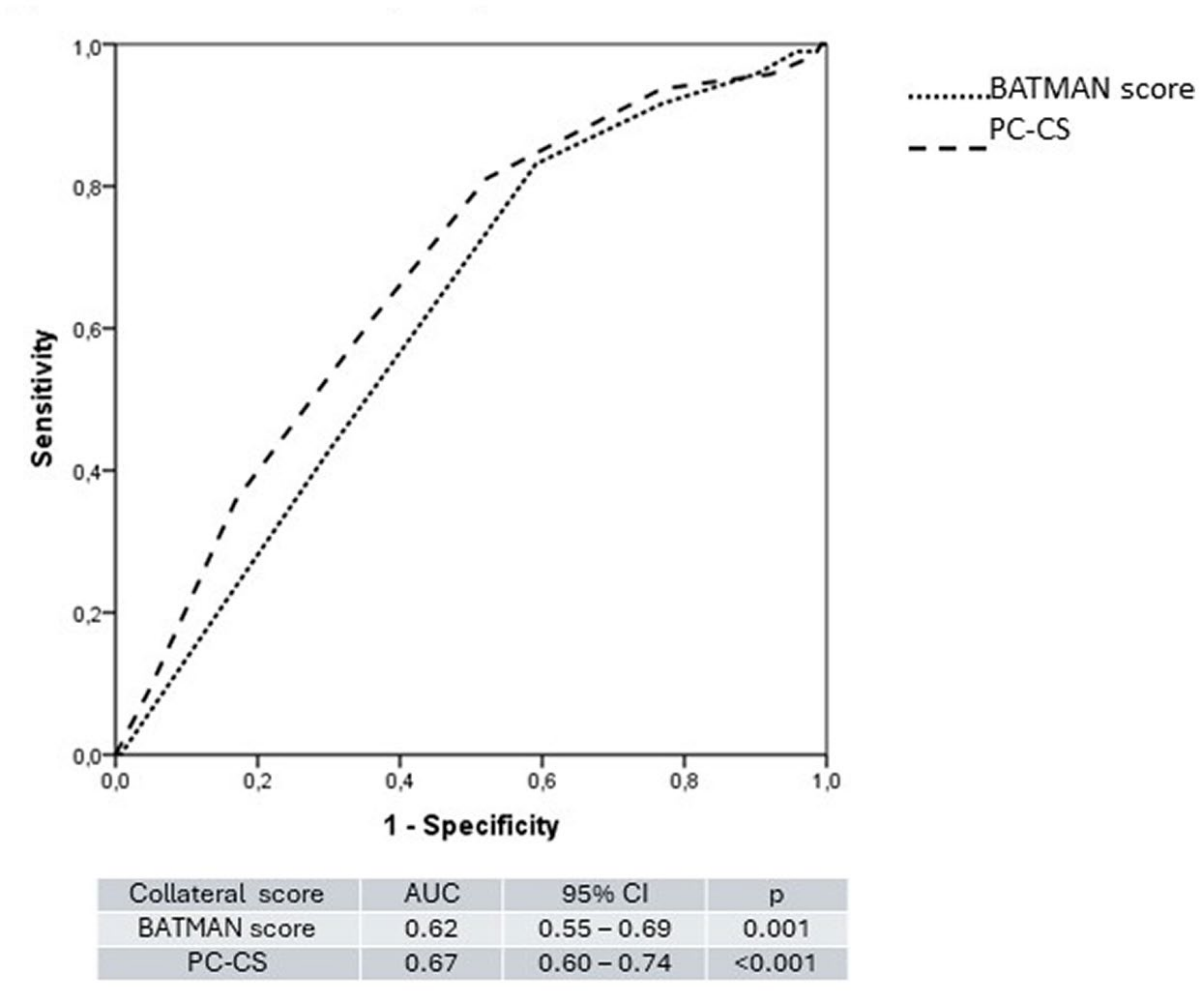
ROC curve comparing the BATMAN score and the PC-CS.

Most patients had an unfavorable outcome at 3 months (177–59%). Higher age (OR 0.97, 95% CI [0.95–0.99], *p* < 0.001) and higher NIHSS (OR 0.97 95% CI [0.95–0.99]; *p* < 0.001) and diabetes (OR 0.43 95% CI [0.24–0.80], *p* = 0.01) were associated with a poor prognosis at 3 months (Table 1). Pc-ASPECTS on baseline NCCT (OR 1.48, 95% CI [1.01–2.17], *p* = 0.04) and length of basilar occlusion (OR 0.94, 95% CI [0.90–0.97], *p* < 0.001) but not pc-ASPECTS on baseline CTA (OR 1.26, 95% CI [1.05–1.52], *p* = 0.15) were significantly associated with the outcome at 3 months. Higher collateral scores were associated with better prognosis at 3 months for both BATMAN (OR 1.45 95% CI [1.16–1.81], *p* = 0.001) and PC-CS (OR 1.44 95% CI [1.22–1.72], *p* < 0.001) (Table 2) but only with a modest accuracy on ROC curve analysis (AUC 0.62, 95% CI [0.55–0.69] and 0.67, 95% CI [0.60–0.74] respectively) (Figure 1). No difference in collateral scores was observed comparing large artery atherosclerosis vs. other etiologies (8 [IQR 7–9] vs. 8 [IQR 7–9], *p* = 0.59 and 7 [IQR 6–8] vs. 7 [IQR 6–9], *p* = 0.23) for the BATMAN and PC-CS, respectively. Patients with poor collaterals had a significant worsening in NCCT PC-ASPECTS at 24 h (PC-CS [median 10 IQR 9–10 VS. 5.5 IQR 4–9; *p* = 0.005] and BATMAN score [median 10; IQR 9–10 vs. 5.5 IQR 3.7–9; *p* = 0.007]) but not in CTA PC-ASPECTS (PC-CS [median 9 IQR 7–10 VS. 8.5 IQR 6.2–10; *p* = 0.11] and BATMAN score [median 9 IQR 7–10 vs. 8.5 IQR 6.2–9.7; *p* = 0.15]).

Higher age and NIHSS were independently associated with an unfavorable outcome at 3 months in both regression models with very similar estimates (OR 0.97, 95% CI [0.95–1.00] and OR 0.91, 95% CI [0.89–0.94]). Endovascular treatment and IV rt-PA thrombectomy were not independently associated a better prognosis although there was a trend for thrombectomy to be associated with better outcome in the PC-CS model (OR 1.77, 95% CI [0.93–3.38], *p* = 0.08). There was also a trend for pc-ASPECTS on NCCT to be associated with the outcome in both models with similar estimates (*p* = 0.06). Both BATMAN score (OR 1.23, 95% CI [0.95–1.60], *p* = 0.11) and PC-CS (OR 1.24, 95% CI [1.02–1.51], *p* = 0.03) showed similar estimates on regression models although only the latter was independently associated with a favorable prognosis at 3 months (Table 3). No effect modification was observed for both collateral scores.

**Table 3:** Logistic regression models with collateral scores.

Characteristics	Model – BATMAN SCORE		Model 1 – PC-CS	
	OR (95% CI)	*p*-value	OR (95% CI)	*p*-value
Age	0.97 (0.95–1.00)	0.03	0.97 (0.95–1.00)	0.05
NIHSS	0.91 (0.89–0.94)	<0.001	0.91 (0.89–0.94)	<0.001
IV rt-PA	1.79 (0.80–4.01)	0.15	1.86 (0.82–4.21)	0.14
Endovascular treatment	1.64 (0.86–3.14)	0.13	1.77 (0.93–3.38)	0.08
pc-ASPECTS (NCCT)	1.54 (0.98–2.40)	0.06	1.52 (0.98–2.35)	0.06
Collateral score	1.23 (0.95–1.60)	0.11	1.24 (1.02–1.51)	0.03

Model with BATMAN score −2 log likelihood: 232.2; Model PC-CS -2 log likelihood: 230.2.

## Discussion

In the present study, both collateral scores were associated with outcomes at 3 months in patients with acute BAO. The two grading systems presented modest prognostic accuracy. Only the PC-CS was independently associated with a favorable outcome at 3 months. Although the BATMAN score did not reach statistical significance, both collateral grading systems performed numerically similar in terms of predicting outcome.

BAO is a disease with high morbidity and mortality. In the present study, patients presented with high NIHSS score (median 21.5, IQR 11–35) and most patients (*N* = 123, 59%) had an unfavorable outcome at 3 months. A similar proportion was observed in previous reports (4, 17). In meta analyses of large series of patients with BAO, the main independent predictors of outcome were the following: stroke severity (assessed with the National Institutes of Health stroke scale), age, location and length of the occlusion, time-to-treatment, recanalization; and collaterals as seen on angiography (4, 18–20). Similarly, in the present study, all these variables, except for time-to-treatment and recanalization, were associated with outcomes at 3 months in the univariable analysis. However, after multivariable analysis, only age, NIHSS and collateral status (PC-CS) remained independently associated with the outcome. NCCT pc-ASPECTS showed a strong trend to be associated with the outcome.

Collateral status is strongly recognized as an important prognostic factor in anterior circulation large vessel occlusion strokes (9, 21). The presence of posterior circulation collaterals in BAO patients is a feature that is still not being regularly used in clinical practice. This is probably related to the more recently proven benefit of mechanical thrombectomy in BAO and also the presence of two different grading systems that involve a more laborious evaluation when compared to the evaluation of leptomeningeal collaterals in the anterior circulation (5, 6).

Retrograde blood flow from the anterior circulation through the posterior communicating arteries (PCoAs) may serve as a primary collateral pathway, potentially providing instant diversion of blood flow to the ischemic occipital and infratentorial regions (22). Secondary collateral pathways comprise arteriolar anastomoses from the inferior and posterior divisions of the middle cerebral artery (MCA), potentially supplying the posterior cerebral artery (PCA) and leptomeningeal anastomoses between the branches of the intradural segments of the vertebral arteries and branches of the basilar artery (23).

The noninvasive nature of CTA and its wide and rapid availability make it an attractive diagnostic modality to assess vascular anatomy in the acute stage and potentially to determine collateral status. The PC-CS allocate more weight on the availability of collateral pathways such as the PICA, AICA, SCA and the PcoA. On the other hand, BATMAN assigns more weight on the clot burden counting the occluded segments of the basilar and vertebral arteries and PCA, also evaluating collateral flow from the PcoA. Previous studies comparing the two grading systems have yielded conflicting results. The PC-CS and BATMAN scores showed a significant association with outcome in previous studies. Alemseged et al. reported the BATMAN score performing better in ROC curve analysis (BATMAN – AUC 0.8; 95% CI [0.7–0.9], vs. PC-CS – AUC, 0.63; 95% CI [0.5–0.7]; *p* = 0.04). Additionally, the PC-CS was not independently associated with outcomes after adjusting for stroke severity (10).

However, in a similar study, Dias et al. reported the PC-CS performing better than BATMAN (AUC 0.80, 95% CI [0.62–0.98] and 0.68, 95% CI [0.47–0.90]) respectively (12). Accordingly, Kwak et al. stated that PC-CS emerged as independent predictor of good clinical outcome, but not BATMAN score. Despite that BATMAN score (area under the curve, 0.701 [95% CI, 0.575–0.827]; *p* = 0.003) and PC-CS (area under the curve, 0.706 [95% CI, 0.583–0.829]; *p* = 0.002) showed a similar overall performance in ROC curve analysis (13).

Different from previous reports, our results show both grading systems to be associated with a better outcome but performing only modestly on ROC curve analysis (AUC 0.67, 95% CI [0.60–0.74] and 0.62 95% CI [0.55–0.69] for the PC-CS and BATMAN score respectively). Novel BAO scores which quantify the amount of thrombus burden in posterior circulation arteries may have greater prognostic accuracy in patients with BAO as it has been shown for patients with anterior circulation stroke with the Clot Burden Score (24).

Our study has limitations. Poor quality admission CTA (34–11.7%) and uncertain/absent BAO (48–16%) were present in a substantial proportion of patients and these were excluded from the analysis. This might have limited statistical power of the analysis. Still, a large number of patients were included achieving a larger sample size than similar previous studies. Even with the use of multivariable analysis, there is still a possibility of residual confounding. However, the PC-CS grading system still predicted a favorable outcome after adjusting for important prognostic factors in BAO (IV rt-PA, endovascular treatment and pc-ASPECTS) including age and NIHSS which are the most important predictors of outcome in stroke. Also, although being applicable to a large proportion of patients, the two grading systems might not perform well enough in certain circumstances such as anatomic variations including fetal variants of the PCA and other persistent carotid-basilar anastomosis.

## Conclusion

Collateral score assessment was significantly associated with outcomes in BAO even when adjusting for important clinical and imaging variables. This emphasizes the importance of collateral assessment in the posterior circulation, analogous to the more established role of collateral assessment in the anterior circulation.

## Data availability statement

The raw data supporting the conclusions of this article will be made available by the authors under reasonable request.

## Ethics statement

The studies involving humans were approved by Conselho Nacional de Ética em Pesquisa (CONEP). CAAE: 66471717.6.2003.5040. The studies were conducted in accordance with the local legislation and institutional requirements. The participants provided their written informed consent to participate in this study.

## Author contributions

FL: Conceptualization, Data curation, Formal analysis, Funding acquisition, Investigation, Methodology, Project administration, Resources, Software, Supervision, Validation, Visualization, Writing – original draft, Writing – review & editing. FR: Investigation, Methodology, Project administration, Visualization, Writing – review & editing. HS: Data curation, Investigation, Project administration, Writing – review & editing. VP: Investigation, Methodology, Project administration, Validation, Writing – review & editing. DD: Investigation, Methodology, Supervision, Validation, Writing – review & editing. IW: Investigation, Validation, Writing – review & editing. CM: Project administration, Visualization, Writing – review & editing, Project administration, Visualization, Writing – review & editing. AY: Data curation, Formal analysis, Investigation, Supervision, Writing – review & editing. WZ: Conceptualization, Methodology, Project administration, Resources, Writing – review & editing. AL: Investigation, Supervision, Writing – review & editing. DB: Investigation, Writing – review & editing. MA: Investigation, Supervision, Writing – review & editing. KB: Formal analysis, Investigation, Writing – review & editing. JG: Investigation, Project administration, Writing – review & editing. LL: Conceptualization, Investigation, Methodology, Validation, Writing – review & editing. WS: Conceptualization, Data curation, Funding acquisition, Investigation, Methodology, Project administration, Resources, Validation, Visualization, Writing – review & editing. OP: Data curation, Funding acquisition, Investigation, Project administration, Resources, Supervision, Validation, Writing – review & editing. FD: Investigation, Project administration, Writing – review & editing. SM: Funding acquisition, Investigation, Project administration, Resources, Writing – review & editing. FM: Conceptualization, Data curation, Formal analysis, Funding acquisition, Investigation, Methodology, Project administration, Resources, Software, Supervision, Validation, Visualization, Writing – original draft, Writing – review & editing.

## Acknowledgments

We would like to acknowledge FAPESP for providing research funding that made possible this trial in Brazil.

## References

[1] Israeli-korn SD Schwammenthal Y Yonash-Kimchi T Bakon M Tsabari R Orion D. Ischemic stroke due to acute basilar artery occlusion: proportion and outcomes. Isr Med Assoc J. (2010) 12:671–5. PMID: 21243866

[2] Voetsch B DeWitt LD Pessin MS Caplan LR. Basilar artery occlusive disease in the new England Medical Center posterior circulation registry. Arch Neurol. (2004) 61:496–504. doi: 10.1001/archneur.61.4.496, PMID: 15096396

[3] Mattle HP Arnold M Lindsberg PJ Schonewille WJ Schroth G. Basilar artery occlusion. Lancet Neurol. (2011) 10:1002–14. doi: 10.1016/S1474-4422(11)70229-0 22014435

[4] Schonewille WJ Wijman CA Michel P Rueckert CM Weimar C Mattle HP. Treatment and outcomes of acute basilar artery occlusion in the basilar artery international cooperation study (BASICS): a prospective registry study. Lancet Neurol. (2009) 8:724–30. doi: 10.1016/S1474-4422(09)70173-5, PMID: 19577962

[5] Tao C Nogueira RG Zhu Y Sun J Han H Yuan G. Trial of endovascular treatment of acute basilar-artery occlusion. N Engl J Med. (2022) 387:1361–72. doi: 10.1056/NEJMoa2206317 36239644

[6] Jovin TG Li C Wu L Wu C Chen J Jiang C. Trial of thrombectomy 6 to 24 hours after stroke due to basilar-artery occlusion. N Engl J Med. (2022) 387:1373–84. doi: 10.1056/NEJMoa2207576, PMID: 36239645

[7] Liu X Dai Q Ye R Zi W Liu Y Wang H. Endovascular treatment versus standard medical treatment for vertebrobasilar artery occlusion (BEST): an open-label, randomised controlled trial. Lancet Neurol. (2020) 19:115–22. doi: 10.1016/S1474-4422(19)30395-3, PMID: 31831388

[8] Alemseged F Nguyen TN Coutts SB Cordonnier C Schonewille WJ Campbell BCV. Endovascular thrombectomy for basilar artery occlusion: translating research findings into clinical practice. Lancet Neurol. (2023) 22:330–7. doi: 10.1016/S1474-4422(22)00483-5, PMID: 36780915

[9] Uniken Venema SM Dankbaar JW van der Lugt A Dippel DWJ van der Worp HB. Cerebral collateral circulation in the era of reperfusion therapies for acute ischemic stroke. Stroke. (2022) 53:3222–34. doi: 10.1161/STROKEAHA.121.037869, PMID: 35938420

[10] Alemseged F Shah DG Diomedi M Sallustio F Bivard A Sharma G. The basilar artery on computed tomography angiography prognostic score for basilar artery occlusion. Stroke. (2017) 48:631–7. doi: 10.1161/STROKEAHA.116.015492, PMID: 28228577

[11] van der Hoeven EJ McVerry F Vos JA Algra A Puetz V Kappelle LJ. Collateral flow predicts outcome after basilar artery occlusion: the posterior circulation collateral score. Int J Stroke. (2016) 11:768–75. doi: 10.1177/1747493016641951, PMID: 27016515

[12] Antunes Dias F Castro-Afonso LH Zanon Zotin MC Alessio-Alves FF Martins Filho RKDV Camilo MR. Collateral scores and outcomes after endovascular treatment for basilar artery occlusion. Cerebrovasc Dis. (2019) 47:285–90. doi: 10.1159/000502083, PMID: 31434074

[13] Kwak HS Park JS. Mechanical thrombectomy in basilar artery occlusion: clinical outcomes related to posterior circulation collateral score. Stroke. (2020) 51:2045–50. doi: 10.1161/STROKEAHA.120.029861 32568658

[14] Langezaal LCM van der Hoeven EJRJ Mont'Alverne FJA de Carvalho JJF Lima FO Dippel DWJ. Endovascular therapy for stroke due to basilar-artery occlusion. N Engl J Med. (2021) 384:1910–20. doi: 10.1056/NEJMoa2030297 34010530

[15] van der Hoeven EJ Schonewille WJ Vos JA Algra A Audebert HJ Berge E. The basilar artery international cooperation study (BASICS): study protocol for a randomised controlled trial. Trials. (2013) 14:200. doi: 10.1186/1745-6215-14-200, PMID: 23835026 PMC3728222

[16] Puetz V Sylaja PN Coutts SB Hill MD Dzialowski I Mueller P. Extent of hypoattenuation on CT angiography source images predicts functional outcome in patients with basilar artery occlusion. Stroke. (2008) 39:2485–90. doi: 10.1161/STROKEAHA.107.511162, PMID: 18617663

[17] Dias FA Alessio-Alves FF Castro-Afonso LH Cougo PT Barreira CMA Camilo MR. Clinical outcomes of patients with acute basilar artery occlusion in Brazil: an observational study. J Stroke Cerebrovasc Dis. (2017) 26:2191–8. doi: 10.1016/j.jstrokecerebrovasdis.2017.04.043, PMID: 28551292

[18] Hacke W Zeumer H Ferbert A Brückmann H del Zoppo GJ. Intra-arterial thrombolytic therapy improves outcome in patients with acute vertebrobasilar occlusive disease. Stroke. (1988) 19:1216–22. doi: 10.1161/01.STR.19.10.1216, PMID: 3176080

[19] Jung S Mono ML Fischer U Galimanis A Findling O De Marchis GM. Three-month and long-term outcomes and their predictors in acute basilar artery occlusion treated with intra-arterial thrombolysis. Stroke. (2011) 42:1946–51. doi: 10.1161/STROKEAHA.110.606038, PMID: 21546481

[20] Arnold M Nedeltchev K Schroth G Baumgartner RW Remonda L Loher TJ. Clinical and radiological predictors of recanalisation and outcome of 40 patients with acute basilar artery occlusion treated with intra-arterial thrombolysis. J Neurol Neurosurg Psychiatry. (2004) 75:857–62. doi: 10.1136/jnnp.2003.020479, PMID: 15146000 PMC1739049

[21] Lima FO Furie KL Silva GS Lev MH Camargo EC Singhal AB. Prognosis of untreated strokes due to anterior circulation proximal intracranial arterial occlusions detected by use of computed tomography angiography. JAMA Neurol. (2014) 71:151–7. doi: 10.1001/jamaneurol.2013.5007, PMID: 24323077

[22] Liebeskind DS. Collateral circulation. Stroke. (2003) 34:2279–84. doi: 10.1161/01.STR.0000086465.41263.06 12881609

[23] Weidner W Crandall P Hanafee W Tomiyasu U. Collateral circulation in the posterior fossa via leptomeningeal anastomoses. Am J Roentgenol Radium Therapy, Nucl Med. (1965) 95:831–6. doi: 10.2214/ajr.95.4.831, PMID: 5321184

[24] Puetz V Dzialowski I Hill MD Subramaniam S Sylaja PN Krol A. Intracranial thrombus extent predicts clinical outcome, final infarct size and hemorrhagic transformation in ischemic stroke: the clot burden score. Int J Stroke. (2008) 3:230–6. doi: 10.1111/j.1747-4949.2008.00221.x, PMID: 18811738

